# Severe mitral regurgitation with bidirectional Coanda effect

**DOI:** 10.1007/s12471-026-02033-x

**Published:** 2026-03-16

**Authors:** Boudewijn Klop, Naomi M. A. J. Timmermans, A. Ramon T. van de Ven, Niels Verberkmoes, Stijn de Ridder

**Affiliations:** 1 Department of Cardiology, Anna Hospital, Geldrop, The Netherlands; 2 Department of Cardio-Thoracic Surgery, Catharina Hospital, Eindhoven, The Netherlands

A 62-year-old man was hospitalized because of weight loss, fatigue, and positive blood cultures with *Streptococcus mitis*. His medical records showed a tricuspid aortic valve with moderate aortic regurgitation and an aneurysm of the abdominal aorta with a diameter of 59 mm, with planned surgery at short notice.

Antibiotic treatment was initiated with intravenous benzylpenicillin, and a transesophageal echocardiogram (TOE) confirmed the diagnosis of infective endocarditis. The TOE showed severe aortic and mitral valve regurgitation, a small sinus of Valsalva abscess, and a vegetation on the anterior mitral valve leaflet. Antibiotic treatment was continued with planned surgery four weeks after the last positive blood culture. However, after 2 weeks of antibiotic treatment, repeat TOE showed progressive disease of the infective endocarditis.

What is the mechanism of mitral regurgitation, and what is its associated Carpentier classification (Fig. 1 and online video supplements A and B)?

**Fig. 1:**
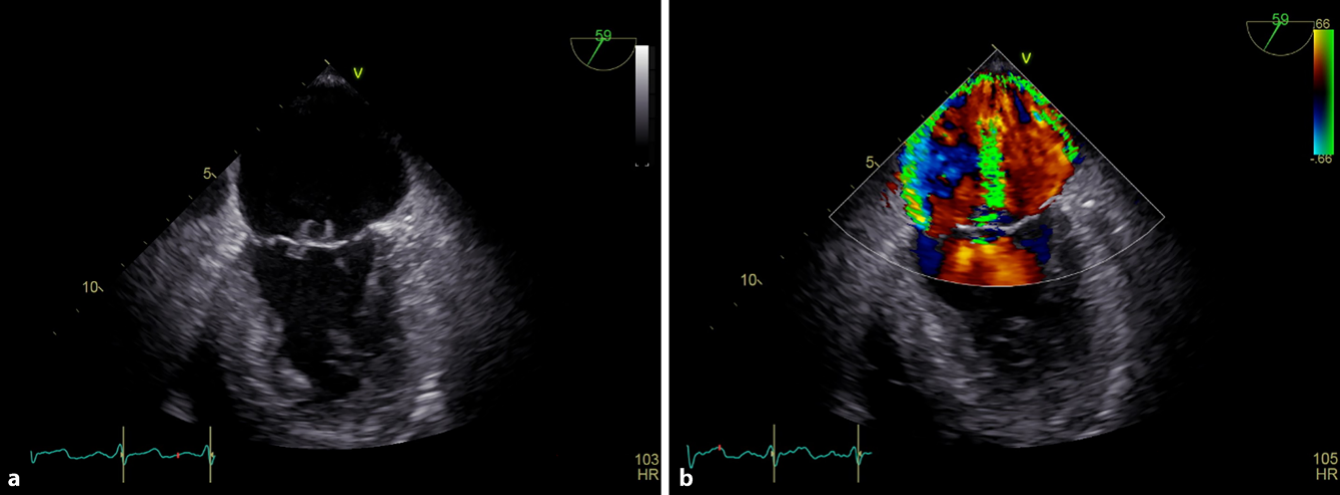
Two-dimensional mid-esophageal commissural view of the mitral valve (**a**) and its concomitant color doppler (**b**)

## Answer

You will find the answer elsewhere in this journal.

## Supplementary Information

**Online video supplement A**: Two-dimensional mid-esophageal commissural view of the mitral valve.

**Online video supplement B**: Two-dimensional mid-esophageal commissural view of the mitral valve with color doppler.

